# Is the Application of Plant Probiotic Bacterial Consortia Always Beneficial for Plants? Exploring Synergies between Rhizobial and Non-Rhizobial Bacteria and Their Effects on Agro-Economically Valuable Crops

**DOI:** 10.3390/life10030024

**Published:** 2020-03-12

**Authors:** Esther Menéndez, Ana Paço

**Affiliations:** MED—Mediterranean Institute for Agriculture, Environment and Development, Institute for Advanced Studies and Research (IIFA), University of Évora, Pólo da Mitra, Ap. 94, 7006-554 Évora, Portugal; apaco@uevora.pt

**Keywords:** sustainable agriculture, plant growth promotion, endophytes, consortium, plant probiotics, field trials

## Abstract

The overgrowth of human population and the demand for high-quality foods necessitate the search for sustainable alternatives to increase crop production. The use of biofertilizers, mostly based on plant probiotic bacteria (PPB), represents a reliable and eco-friendly solution. This heterogeneous group of bacteria possesses many features with positive effects on plants; however, how these bacteria with each other and with the environment when released into a field has still barely been studied. In this review, we focused on the diversity of root endophytic rhizobial and non-rhizobial bacteria existing within plant root tissues, and also on their potential applications as consortia exerting benefits for plants and the environment. We demonstrated the benefits of using bacterial inoculant consortia instead of single-strain inoculants. We then critically discussed several considerations that farmers, companies, governments, and the scientific community should take into account when a biofertilizer based on those PPBs is proposed, including (i) a proper taxonomic identification, (ii) the characterization of the beneficial features of PPB strains, and (iii) the ecological impacts on plants, environment, and plant/soil microbiomes. Overall, the success of a PPB consortium depends on many factors that must be considered and analyzed before its application as a biofertilizer in an agricultural system.

## 1. Why Do We Need More Sustainable Agriculture?

Currently, the global population is continuing to grow and is causing noticeable environmental damage, mostly driven by the misuse of natural resources due to the large ecological footprint of the humankind. According to the Food and Agriculture Organization (FAO) of the United Nations, the population will reach an unprecedented size in the coming years, mostly due to the high life expectancy in developed countries and to the high fertility of younger generations in developing and underdeveloped countries [1]. This situation makes increasing the food supply and a more equitable distribution of these food resources one of the prime concerns of our society [2,3].

Agriculture, which represents the primary sector globally and is crucial to feeding the population and livestock, covers more than one third of the world’s available land in total [1]. Even if more land could be dedicated to agriculture or used to create more intensive agricultural systems, there is no benefit to doing so, due to the severe negative effects that this land expansion and agricultural intensification might have [4]. In order to increase crop productivity, it is a common practice for farmers to increase the use of chemical fertilizers, namely nitrogen, phosphate, or potash fertilizers. In fact, the development of the technology for the production of chemical fertilizers in the 1930s to 1950s, as well as other technical improvements in agriculture driven by governments and companies, led to the so-called “Green Revolution” during the 1960s [4,5]. This “Green Revolution” created many advantages all over the world; however, in the long term, it brought negative consequences and disadvantages that are apparent today.

**Figure 1:**
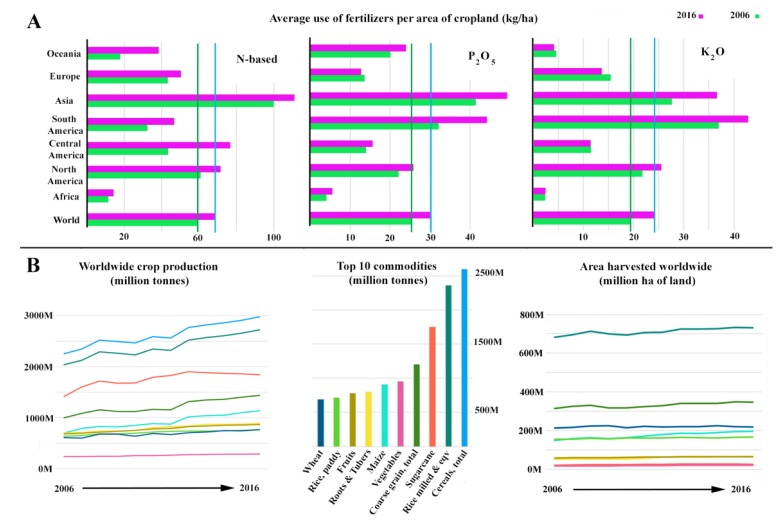
Graphical representation of data available on FAOSTAT. (**A**) Average use of fertilizers per area of cropland (kg/ha): from left to right, data regarding to N-based, phosphate, and potash, all of which are synthetic fertilizers. Graph shows the difference between 2006 and 2016 in broad areas of the World, as well as the average use in 2006 and 2016 worldwide. (**B**) Data regarding to the most produced commodities all over the world and the area harvested worldwide.

Based on the available data from FAOSTAT (Statistic Division of the Food and Agriculture Organization of the United Nations), in 2017, the five countries using the most nitrogen-based fertilizers (for agricultural use, in million (M) tonnes) were China (29.62 M), United States of America (USA; 11.58 M), Indonesia (2.95 M), Canada (2.47 M), and Vietnam (1.69 M). China and the USA were also the countries with the highest use of P-(P_2_O_5_) (15.37 M and 4.27 M. respectively) and K (K_2_O)-based fertilizers (13.59 M and 4.79 M, respectively). Canada, Australia, Malaysia, Indonesia, and Vietnam also appeared within the top 10 countries using these kinds of chemical fertilization [1]. According to the FAOSTAT database, there was an increase in the use of N-, P-, and K-based fertilizers between 2006 and 2016 in every area in the world (Figure 1A). The average use of chemical fertilizers per unit area of cropland is reaching an especially alarming level in Asia and America (both North and South America).

Sugarcane, grain legumes, maize, wheat, rice, and vegetables are included in the top 10 commodities worldwide, representing the major part of crop production and occupying most of the arable land available, with production increasing significantly in some cases in recent years (Figure 1B). Moreover, other crops such as quinoa, ginger, and rapeseed (oilseeds) are reaching significant levels in terms of crop establishment [6,7], leading to higher productions and more arable land being covered with these crops, replacing other less “profitable” ones. Interestingly, the area harvested and the land area dedicated to major crops did not increase during the 2006–2016 period. These facts may suggest that the increase in crop production was related mostly to the application of fertilizers, although there are other factors to consider that might have contributed, such as the use of crop varieties with improved features (use of stress-tolerant varieties and/or naturally adapted or genetically modified (GMO) crops, amongst others) [8,9,10].

**Figure 2:**
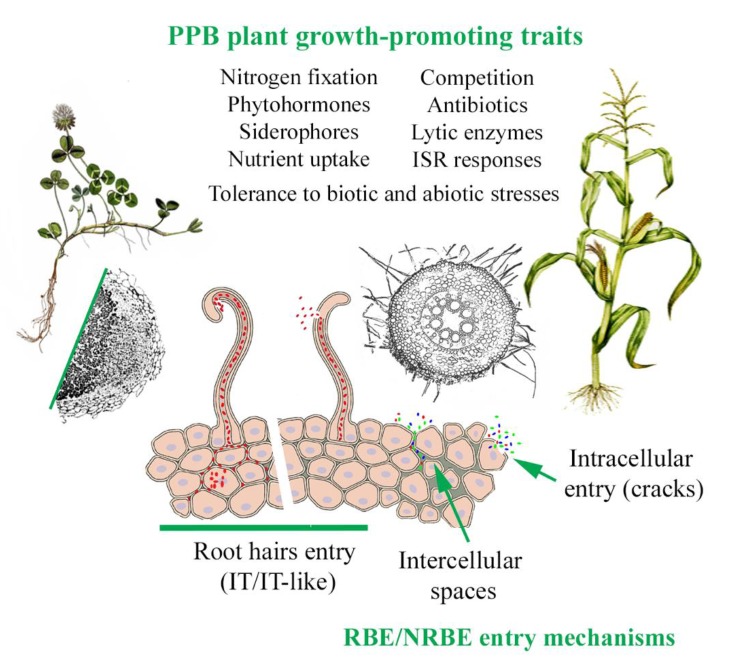
Plant-growth-promoting attributes and entry mechanisms showed by plant probiotic bacteria (PPBs) on plants. Both RBEs (rhizobial bacterial endophytes) and NRBEs (non-rhizobial bacterial endophytes) can exert one or several plant-growth-promoting traits. The success in the colonization and infection of these bacteria will result in a better plant performance. The root hair entry mechanism is usually associated with RBEs. NRBEs might also enter actively by root hairs, by just erode the cell wall, forming (or not) a tubular structure similar to an infection thread (IT), or passively, via the IT formed by rhizobia. Both RBEs and NRBEs might also enter actively or passively by the other mechanisms described in the figure.

The application of chemical fertilizers is causing harmful effects to the environment, such as soil acidification, water eutrophication, and air pollution, amongst other problems [3,11]. Thus, it is becoming crucial to achieve higher production through sustainable practices, avoiding or at least reducing the use of chemical fertilizers. The use of “greener” fertilizers, namely biofertilizers, mostly based on plant probiotic bacteria (PPB), represents a solution to most of the problems that result from the application of chemical fertilizers [12]. This heterogeneous group of beneficial bacteria has many features with positive effects on plants (Figure 2). Within the PPBs, there are bacteria able to perform biological nitrogen fixation (BNF), known as nitrogen-fixing bacteria, others capable of solubilizing P and K forms that are not available to plants, known as P- or K-solubilizing bacteria (PSB and KSB, respectively). Moreover, the ability to biosynthesize phytohormones and siderophores are two of the most common plant-growth-promoting mechanisms displayed by PPBs [13,14,15]. Furthermore, PPBs also have biocontrol properties, acting as plant-protecting agents against pathogens of different nature. The production of siderophores and cell-wall-hydrolytic enzymes (also considered plant growth promotion mechanisms), as well as the emission of volatile compounds (VOCs) and the production of antimicrobial compounds, are among those biocontrol properties [16]. The use of PPBs not only improves crop production, but also the quality of the grains, fruits, or products derived from these crops [17].

The potential use of these beneficial bacteria in biofertilization schemes has caught the eye of the society in general. Farmers need to obtain high yields of healthier crops in response to the increased food demand of consumers [4]. Moreover, governments have the responsibility to create policies governing the use of sustainable and eco-friendly fertilizers [4]. Biotech companies such as Bayer, Novozymes, and BASF, amongst others, develop and commercialize biofertilizers based on PPBs, this being a blooming and profitable market [4]. The production and application of single and mixed inoculants based on selected plant probiotic bacteria is now very attractive at both economic and ecological levels, representing a real alternative for the sustainable reduction of the use of synthetic fertilizers in fields [12,18].

## 2. The Plant Microbiome, with a Focus on the Roots and Endophytic Plant Probiotic Bacteria

Plants harbor a complex microbiome within their inner tissues, which can be considered a second genome. A plant without any associated microbiota is very rare in nature [19]. Plants and their microbiomes can behave as one entity, which has also close relationships with the environment, namely with the so-called rhizosphere microbiome [20,21]. The term coined to refer to this “intimate partnership” is the plant holobiont [22]. The scientific community is invested in the understanding on how the plant holobiont works, where microbiomes come from, and how to manage them in order to obtain some profit for agricultural systems [23,24,25,26,27,28,29,30,31,32].

Here, we focused on a subset of the plant microbiome: the bacteria displaying endophytic behavior from rhizobial (rhizobial bacterial endophytes; RBE) and non-rhizobial (non-rhizobial bacterial endophytes; NRBE) origins; these are also considered plant probiotic bacteria, since they promote plant growth using different mechanisms. This group of endophytic bacteria is very diverse and heterogeneous, and members commonly belong to the phyla Proteobacteria, Firmicutes, Actinobacteria, and Bacteroidetes [23]. These bacteria easily enter plant roots through different entry mechanisms (Figure 2), such as intracellular invasion, crack entry, or root hair invasion, the latter being commonly associated with rhizobial species entering legume roots [33,34]. Although the presence of plant probiotic endophytic bacteria in shoots, leaves, flowers, and seeds of plants is also regularly studied [35], in this review we focused on the root endophytic bacteria; some such endophytes might even travel actively or passively to the upper parts of the plants via vascular tissues [36,37].

In this work, have recurrently mentioned the microbiome from legume root nodules, because besides accommodating rhizobia strains, these nodules can also be occupied by diverse non-rhizobial bacteria [38,39,40]. Interestingly, there have been some works proposing the microbiomes of legume nodules to be key sources of endophytic PPBs (RBEs and NRBEs), claiming that this particular legume organ harbors a wide range of beneficial endophytes with plant growth promotion features that fulfil biosafety rules [40,41,42].

## 3. Plant Probiotic Bacteria with Potential for Improving Crop Yields: Which Are They and How Do They Promote Plant Growth?

### 3.1. What Are the RBEs?

Rhizobia are a complex group of Gram-negative bacteria that live in the soil as saprophytes or in association with plants, as endophytes of inner plant tissues. These bacteria have the amazing availability to fix atmospheric nitrogen when located within the nodule and to convert this nitrogen into available nitrogen compounds to be used directly by the legumes, these features having been extensively studied for decades [43,44]. As well as this ability, rhizobia also possess further features that are beneficial for plant development, whether in free-living conditions (rhizosphere) or forming biofilms in the rhizoplane or in association with plants as endophytes [45,46]. Moreover, rhizobia are able to protect the plant host against pathogens, functioning as biocontrol agents [47,48,49].

Recent studies have identified the orders Rhizobiales (α-rhizobia), and Burkholderiales (ß-rhizobia) as keystone taxa, since they are always present in the core microbiomes of plants. The genera *Rhizobium* and *Bradyrhizobium*, belonging to the order Rhizobiales, and the genus *Burkholderia*, belonging to the order Burkholderiales, are the ones with better-known functions in improving the plant productivity and maintaining community evenness [50,51].

There is a plethora of previous work showing the beneficial effects of rhizobial strains on the growth and development of several non-legume crops, such as sorghum, lettuce, carrots, spinach, tomato, pepper, strawberries, barley, wheat, rice, and cotton, amongst others, improving in most of these crops the quality of both the edible and non-edible parts [52,53,54,55,56,57,58,59,60]. The majority of these studies have shown the effects of these interactions only under laboratory- or greenhouse-controlled conditions.

### 3.2. What Are the NRBEs?

The beneficial non-rhizobial bacterial endophytes are taxonomically very diverse, and their functions are commonly associated with their plant-growth-promoting activities. They are also known as “helpers”, improving the effectiveness of the rhizobium–legume symbiosis [61,62].

Pseudomonadales, Enterobacteriales, and some Bacilli and Paenibacilli are among the NRBE taxa most commonly found in below-ground plant structures, whether the approaches analyzing microbiomes are culture-dependent or culture-independent. As an example of a culture-dependent study, Brígido et al. [63] showed that the genera *Enterobacter* and *Pseudomonas* were the most frequently identified genera in chickpea roots. Moreover, Trujillo et al. [64] identified the genus *Micromonospora* as being commonly associated with legume nodules, and Muresu et al. [65] showed that the NRBE-populated nodules of several legumes included at least the genera *Bacillus*, *Pseudomonas*, *Xanthomonas*, *Agromyces*, *Microbacterium*, *Curtobacterium*, and *Micromonospora*, and members of the family Enterobacteriaceae. Interestingly, de Meyer et al. [66] reported the concurrence of some rhizobia with some of the identified NRBEs, which belonged to the genera *Pseudomonas*, *Bacillus*, and *Paenibacillus*, among the most frequently isolated NRBEs from the nodules of 30 different leguminous plants.

In culture-independent studies, apart from the already stated Rhizobiales, commonly found in root microbiomes [46], the orders Sphingomonadales, Enterobacteriales, Burkholderiales (NRBE), Caulobacterales, and Rhodospirillales, from the phylum Proteobacteria, and the genus *Syntrophomonas* from the phylum Firmicutes are the most abundant in *Trifolium* entire roots [67]. Proteobacteria and Firmicutes are the phyla with the highest operational taxonomic units (OTU) abundancies in the rice root endosphere microbiome [68]. In maize roots, the phylum Proteobacteria accounts for approximately the 85% of the total root microbiome [69]. In this study, the family Enterobacteriaceae and the genera *Burkholderia* (we denoted the latter genus as NRBE, but its symbiotic members were considered ß-rhizobia [70]), *Herbaspirillum*, *Curvibacter*, *Acinetobacter*, *Stenotrophomonas*, and *Pseudomonas* (Proteobacteria) and *Curtobacterium* (Actinobacteria) were significantly enriched within maize roots.

Overall, the genus *Pseudomonas* and its closely related genera seem to be the most abundant genera in root microbiomes analyzed by both culture-dependent and -independent approaches. The members of this genus have been extensively studied and display a complete range of beneficial traits for plants. For instance, Hu et al. [71] suggested that probiotic *Pseudomonas* communities may manage the functionality of the microbial communities associated with tomato via regulation of important plant-growth-promoting traits. Moreover, some authors have attributed the capacity to form nodules in legumes to some strains of this genus, as well as to some *Paenibacillus* and Enterobacteria strains [40,72,73,74]. However, these studies must be taken very cautiously, as they showed no conclusive proof or correct controls. The affirmation of the nodulation capacity attributed to these strains must be supported with tests that provide unequivocal proof of this particular ability [75].

### 3.3. How Do They Promote Plant Growth?

There are many reviews offering an overview of the vast information about microorganisms, and specially bacteria, that are plant growth promoters and biocontrol agents, and also reviewing the mechanisms associated with these particular functions, as well as more publications showing and/or reviewing the benefits to plants, such as the enhancement of bioactive compounds in the edible parts, among others [3,12,13,14,15,76,77,78,79,80,81,82,83].

Briefly, PPBs have features involved in the (i) facilitation of nutrient acquisition, (ii) production of phytohormones and modulation of their levels, (iii) tolerance to either abiotic or biotic stresses, (iv) production of siderophores and other metabolites, and (v) induction of disease resistance, among other properties not listed here (Figure 2). The expression of these promotion activities when RBEs and NRBEs are in association outside or inside the plant tissues will make the difference in most cases with a single inoculation of either RBEs or NRBEs separately.

## 4. Better Together—Inoculation with PPB Consortia vs. Individual PPBs

Nowadays, it is accepted that bacteria function mostly as consortia in diverse natural microbiomes. However, the molecular basis of plant–NRBE/RBE associations remains poorly understood, at least compared to what is known about the symbiotic relationships between legumes and rhizobia or plants and their pathogenic bacteria [78,84]. These relationships that occur between plants and microbes have high levels of complexity. The addition of a given bacterial consortium will increase the complexity of those systems, more so than if only a single strain is inoculated [85].

Many studies have reported co-inoculation assays of NRBE and RBE strains in plants, and have proven that inoculating PPBs in consortia provides many benefits to plants [86,87,88,89,90]. However, most of these studies were performed in lab- and greenhouse-controlled conditions, only a small proportion having been performed under field conditions (Table 1 and references cited therein). In fact, the improvement of symbiotic effectiveness of RBE by co-inoculation with other beneficial NRBEs [91,92,93,94,95], as well as other RBEs forming a consortium [96,97] are commonly observed showing that the strains might assume one or functions depending on many factors. Moreover, there are studies combining RBE/NRBEs with mycorrhiza, showing improved results in yields and quality, as well as reducing the amount of chemical fertilization [98,99,100,101,102,103].

In the particular case of legumes, RBEs and NRBEs living within nodules certainly act synergistically to increase plant growth, health, and survival [40,41]. Interestingly, some studies show that the taxonomic composition of the nodule microbiome varies depending on which rhizobium has induced the nodules. For instance, Lu et al. [99] showed significant differences in rhizosphere and nodule bacterial communities when *Dalbergia* plants were nodulated with *Rhizobium, Bradyrhizobium*, or *Burkholderia* strains. Dominant OTUs found were *Lactococcus*, *Pseudomonas*, *Bacillus*, and Cyanobacteria. Moreover, these authors found differences in relative abundances when nodules were formed in N-fed or N-limited conditions. The benefits of RBEs and NRBEs associations are not restricted to legumes, extending also to non-legume species. For example, the co-inoculation of *Bacillus* and *Pseudomonas* (two NRBEs) in tomato plants increased yield and enhanced the nutrient content of tomato fruits [117]. Interestingly, the inoculation of a given NRBE strain (*Bacillus*) did not alter bacterial communities associated with tomato roots in the long term [118], which would be good in agricultural systems for the maintenance of native microbiomes, because the microbial community will absorb the added strain after this strain produces the beneficial effect to the plant.

Taking all the above into account, we may assume that the benefits of mixed inoculation happen due to the combination of plant-growth-promoting activities and, in the case of the combination of RBEs and NRBEs, the non-rhizobial bacteria act to favor functions clearly attributed to rhizobia, such as the nodulation and nitrogen fixation. Therefore, plant co-inoculations with RBE and NRBE consortia have benefits in the development of both legume and non-legume plants, which highlights that these consortia are efficient biofertilizers for use in the future, in a more sustainable mode of agriculture. Nevertheless, there is no magical combination, since the beneficial effects shown in nodulation and yield when a certain non-rhizobial strain is co-inoculated with a rhizobial strain may not display the same beneficial effect in another crop, or may not produce significant increases as it is expected. For instance, Camacho et al. [119] showed that a *Bacillus* strain, considered a proper PGPR, was beneficial in the *R. tropici–Phaseolus vulgaris* symbiosis, but had deleterious effects when interacting with the *B. japonicum–Glycine max* relationship. Table 1 also shows some other examples in which the effects on plant growth and yield derived from inoculation of PPB consortia in field tests were not always beneficial or did not produce significant increases of yield or quality [87,88,110,111,112,113,114,115]. These negative findings might be due to the enormous variety of factors affecting crops, especially in the field, where microbiomes and plants are subjected to biotic and abiotic stresses. The use of different “omics” techniques, including metagenomics or transcriptomics, will elucidate which are the mechanisms underlying both beneficial and deleterious effects. Thus, there is a need for future work on several aspects and considerations, which are discussed in the next section.

## 5. Considerations for More Efficient Use of PPB Consortia in the Fields

An overwhelming number of studies has been published about the isolation of new plant-growth-promoting bacteria, some of which showed outstanding benefits for plant growth. Moreover, as the omics techniques, such as metagenomics or genome sequencing, are becoming more economically accessible for to scientific community (companies are offering cheaper prices per sample), the number of works deciphering plant and soil microbiomes is increasing [120]. Undoubtedly, knowledge is of paramount importance, but it is necessary to acquire a deeper understanding about the molecular dialogues among plants, microbes, and how these interactions are shaped by the environment. Moreover, not all bacteria detected by metagenomics and other omics might be suitable for commercial inoculants because they could be impossible or difficult to grow in standard laboratory conditions [121,122].

There are research areas that must be prioritized, such as the study of crop microbiomes globally, the development of host–microbiome models to study functions and possible interactions, and abandoning the idea of a single solution that will fit all cases [27,40]. For instance, Kong et al. [123] proposed the use of simplified microbial consortia (SMCs) in order to improve crop yields. The microbial components of such SMCs should be selected from the core microbiomes and the rhizosphere microbiomes of crops through omics approaches. Synergies among SMC components, plant growth promoting activities, and changes in natural population dynamics induced by them should then be tested. Apart from this “microbial modeling” for the design of a successful biofertilizer composed of a given consortium, other authors have also considered the formulation and the appropriateness of the carrier/delivery system to the crops as key points to take consider [124]. Here, we focused on some of the points that we think are currently less discussed, and that we consider to be priorities: a reliable taxonomic and functional identification of the strains forming part of a consortium with possibilities of application, and the ecological constraints that will appear upon inoculation of bacteria consortia to improve crop yields.

### 5.1. Reliable Identification and Characterization of Inoculant Strains

Bashan et al. [125] proposed a series of rules that authors of new manuscripts about plant protection products should follow, including taxonomy rules, formulation content, and details of the application methods. Some authors are reluctant to correctly identify bacterial strains, arguing that their studies are not about bacterial taxonomy and/or systematics. The correct taxonomic identification of the given strains that are present or not in a consortium must be resolved from the beginning of a particular study. A correct identification must also be performed when fungal or algal species are included in the formulations. Misidentification of strains and problems regarding taxonomical affiliations are more common than should be allowed. Why do we need to identify our isolates correctly? It is all about biosafety and environmental awareness. For instance, some of the examples given in Table 1, such as the inoculation of crops with strains of *Pseudomonas aeruginosa* [108], among others, might represent a biosafety conflict, as many strains belonging to these species are pathogenic to humans and animals.

In recent decades, the biofertilizer market has grown exponentially, and governments have enacted many policies and laws concerning the marketing and application of these products [4,126]. Companies and scientists developing such products are required to follow these biosafety rules. Some countries are more permissive, some countries are very strict, and some of them do not have policies regulating these products yet. Big chemical fertilizer companies as well as new SMEs are now interested in the production and marketing of microbe-based products [127]. For example, the European Commission (EC) has approved regulations and policies governing the use of biofertilizers based on microorganisms; however, these policies are restrictive in terms of the permitted microorganisms, maybe due to the presence of strong fertilizer industries [127]. In June 2019, the EC enacted the “Regulation (EU) 2019/1009 of the European Parliament and of the Council of 5 June 2019 laying down rules on the making available on the market of EU fertilizing products”, which amended Regulations (EC) No 1069/2009 and (EC) No 1107/2009 and repealed Regulation (EC) No 2003/2003. This Regulation has a section in which only four kinds of microorganism are acceptable as part of a biofertilizer: *Azotobacter* spp., *Azospirillum* spp., *Rhizobium* spp. and mycorrhiza (Annex II). Nevertheless, Article 42 “Amendment of Annexes” provides the basis for adding more microorganisms to the list of those allowed. This article contains a checklist that must be completed before the registration of a PPB-based product, including the correct and complete taxonomical classification of the microorganisms, as well as proofs confirming these microorganisms to be “Generally Recognized as Safe” (GRAS).

### 5.2. Ecological Constraints that Might Affect Inoculant Functioning in the Field

Most studies describing RBE and NRBE consortia are still performed under controlled conditions in labs and greenhouses, without any further study about the ecological implications or survival under field conditions [128]. The effects produced among components of a consortium and/or between single or mixed bacteria with a plant depend mostly on biotic and abiotic factors [19]. The effect of a bacterium or a consortium might be specific or restricted to some conditions as well as their establishment as endophytes; some strains or mixes of strains do not always achieve the desired endophytic lifestyle. For instance, different strains of the genus *Bacillus* have different effects when inoculated on broccoli sprouts under field conditions. These strains did not establish themselves as endophytes, but some of them promoted plant growth promotion and influenced the bacterial communities [129]. Moreover, interactions with other microorganisms in the soils and plant tissues, such as fungi, oomycetes, and other bacteria, must be considered. For example, the PPB strains of the genus *Azospirillum* showed good plant-growth-promoting attributes but a less good capacity to compete with other bacteria associated with the plants [130].

The ecological impact generated when a field is inoculated with a PPB-based consortium must be addressed before the inoculation. For example, the addition of either N fertilizers or *Rhizobium*-based fertilizers significantly reduced the OTU richness and relative abundances in rice roots [131]. In this work, the rice–endophytic community presented genera *Rheinheimera*, unclassified Rhodospirillaceae, *Pseudomonas*, *Asticcacaulis*, *Sphingomonas*, and *Rhizobium* as dominant taxa. This community may have been influenced by the positive synergistic impact of rhizobial biofertilizer inoculation (with one strain belonging to the genus *Rhizobium*) in combination with low N fertilization. Indeed, the combined treatment showed the greatest vegetative growth promotion. Although this study was performed in a greenhouse, the use of soils directly extracted from the cultivation fields meant that the obtained results were closer to reality.

Interestingly, it is generally assumed that the inoculation of free-living diazotrophic bacteria has less impact on soil and plant microbiomes than the inoculation of rhizobial endosymbionts, because of the symbiotic specialization of the latter group [130]. The success of a given PPB consortium/biofertilizer relies on its specific interactions with the plants, which are mediated by plant exudates and the ability of the PPBs to compete with other microbes [130]. If the PPBs are true beneficial endophytes and are able to colonize plant roots, they will be capable of altering the natural microbial community. This fact could lead to a two-sided effect, which might be good or bad for the communities, depending on what, how, and when the effects are evaluated.

## 6. Conclusions

The use of plant probiotic RBEs and NRBEs as inoculants has the potential to increase crop yield without the overapplication of chemical fertilizers, pesticides, and fungicides, and consequently to reduce the environmental impact in agriculture and maximize the production of heathier and safer foods. RBEs and NRBEs establish synergies and act complementarily when forming a consortium, providing beneficial effects to crops. Although a high number of these associations are well-documented and most of them show positive results, there is still a lack of knowledge on how these consortia behave and interact with plants, environments, and other microbes (the microbiome). Nonetheless, there are many consortia already being commercially exploited. We may conclude that the use of these beneficial bacteria combined in consortia is very important for improving crop yields and performance. However, the overall ecological impact is still not well-known and must be addressed in future studies if the successful and sustainable application of bacterial consortia is to be pursued for a better, greener, and more profitable agriculture.

## Figures and Tables

**Table 1:** Examples of plant probiotic bacteria applied as consortia and tested on field conditions.

Consortium Composition	Plant Host and Conditions	Effects on Tested Crops	Reference
**RBE + NRBE**			
*Bradyrhizobium japonicum + Rhizobium tropici +Azospirillum brasilense*	Soybean and common bean field trials in Brazil. *Azospirillum* applied in-furrow, rhizobia applied on seeds.	Increases in grain yield: 16.1% soybean/19.6% common bean respective to indigenous rhizobial population, and 7.1% soybean/14.7% common bean respective to rhizobia-inoculated treatment.	[104]
*Rhizobium* and 2 *Bacillus (B. subtilis + B. megaterium)*	Common bean fields trials in Eastern Anatolia	No significant effect on common bean yield compared with single inoculations.	[105]
*R. leguminosarum + Bacillus sp. + Pseudomonas sp.*	*Phaseolus vulgaris*/two field trials	Average grain yield was enhanced by 39% after application of consortium.	[106]
*R. leguminosarum +* *P. fluorescens*	Lentil field trials (2) with different P fertilization levels	Improved symbiotic parameters, leghemoglobin content, growth, and grain yield of lentil with 50% P reduction.	[107]
*Bradyrhizobium + P. aeruginosa*	Soybean field assay	Improved plant growth but not to a statistically significant degree. Nodule weight was significantly higher in the co-inoculated soybean treatment.	[108]
*Mesorhizobium* + *P. fluorescens* or *P. aeruginosa/* *Azotobacter chroococcum/* *B. megaterium*	Chickpea field assays	Improvements depended on the partner PGPR. Combination with *Pseudomonas* showed best results.	[88]
*Rhizobium + Sinorhizobium +* *Bacillus + Burkholderia*	Pidgeon pea (*Cajanus cajan*) field trial	Increased plant biomass and nodule mass per plant.	[93]
**NRBE**			
*Arthrobacter nitroguajacolicus + B. cereus + B. megaterium + B. mojavensis + P. azotoformans + P. frederiksbergensis*	Two field plots of *Nicotiana attenuata* with fungal disease problems	Significantly reduced disease incidence and mortality in the infected field plot without influencing growth or herbivore resistance	[109]
Mixes of various *Pseudomonas, Enterobacter and Serratia strains*	Rapeseed field trials (also greenhouse trials)	Not statistically significant increases of rapeseed oil and grain yields	[110]
*Pseudomonas sp. + Chryseobacterium sp.*	Rice, paddy. Three cropping seasons of a rice field	Significantly increased rice production and enhanced, but not to a significant degree, the quality of the rice grains. Protected against rice blast fungus.	[111]
Various consortia involving *Enterobacter, Serratia, Pseudomonas, Microbacterium* and *Achromobacter*	Avocado seedlings grown in a nursery located outdoors and within a commercial avocado orchard	Mitigate water shortage and salt stress. No report on plant biomass or yield increments.	[112]
*Rhodotorula graminis + Rahnella sp. + Burkholderia sp. + Acinetobacter calcoaceticus + R. tropici + Sphingomonas yanoikuyae + P. putida + Sphingomonas sp.*	Production of Douglas fir plantlets in a nursery	Not significant increases in weight of shoot and N content in needles.	[113]
**RBE**			
2 *Burkholderia sp.*	Fenugreek field trial	The grain yield was enhanced by 40% as compared to control in field trials. Inhibited *Fusarium* spp.	[114]
*Occhrobactrum ciceri + Mesorhizobium cicero*	Different kabuli and desi type chickpea genotypes in a field trial in two different soils	One Desi genotype showed increased nodulation in plants co-inoculated. Increased biomass and grain yield in every genotype.	[115]
Various consortia of 2 *R. leguminosarum bv trifolii strains*	Extensive rice field trials in the Egyptian Nile Delta	Significant increases in straw biomass. Reduction of the use of synthetic N fertilizers.	[116]
**RBE/NRBE with Mycorrhiza**			
2 *Pseudomonas* + mixed mycorrhiza	Open field trial in a real industrial tomato farm	Increases of flowering, dimensions, and weight of tomato fruits and improved industrial and nutritional features of fruits.	[100]
Commercialized consortia of *Azotobacter vinelandii* and Mycorrhyza *(Rhizophagus irregularis)*	Common wheat field trials with different NPK fertilization rates	When inoculated with medium and high fertilization, promoted cluster shift according to vectors/variables related to root growth and nutrient allocation in the grains and limits root growth at low fertilization levels.	[101]
*Acinetobacter sp., Rahnella aquatilis,* 2 strains of *Ensifer meliloti* and *Glomus + Sclerocystis + Acaulospora*	Wheat and *Vicia faba* field trials	Application of either only PGPB alone or PGPB + mycorrhyza increased parameters.	[102]
*P. fluorescens + Azospirillum lipoferum* + *Glomus intrarradices*	Maize field trials. Fertilized, reduced, and not fertilized.	No increase in production or plant biomass. Restored production with the combination 50% fertilizer + consortium.	[103]

## References

[1] FAO (2018). World Food and Agriculture—Statistical Pocketbook.

[2] Ehrlich P.R., Harte J. (2015). Opinion: To feed the world in 2050 will require a global revolution. Proc. Natl. Acad. Sci. USA.

[3] Gouda S.K., Saranga H. (2018). Sustainable supply chains for supply chain sustainability: Impact of sustainability efforts on supply chain risk. Int. J. Prod. Res..

[4] García-Fraile P., Menéndez E., Celador-Lera L., Díez-Méndez A., Jiménez-Gómez A., Marcos-García M., Cruz-Gonzalez X.A., Martinez-Hidalgo P., Mateos P.F., Rivas R., Kumar V., Shivesh M.K., Prasad S. (2017). Bacterial Probiotics: A truly green revolution. Probiotics and Plant Health.

[5] Pingali P. (2012). Green revolution: Impacts, limits, and the path ahead. Proc. Natl. Acad. Sci. USA.

[6] Borras S.M., Franco J., Isakson S.R., Levidow L., Vervest P. (2016). The rise of flex crops and commodities: Implications for research. J. Peasant Stud..

[7] Bazile D., Jacobsen S.E., Verniau A. (2016). The global expansion of quinoa: Trends and limits. Front. Plant Sci..

[8] Tester M., Langridge P. (2010). Breeding technologies to increase crop production in a changing world. Science.

[9] Nuccio M.L., Paul M., Bate N.J., Cohn J., Cutler S.R. (2018). Where are the drought tolerant crops? An assessment of more than two decades of plant biotechnology effort in crop improvement. Plant Sci..

[10] Paul M.J., Nuccio M.L., Basu S.S. (2018). Are GM crops for yield and resilience possible?. Trends Plant Sci..

[11] Savci S. (2012). An agricultural pollutant: Chemical fertilizer. Int. J. Environ. Sci. Dev..

[12] Menendez E., Garcia-Fraile P. (2017). Plant probiotic bacteria: Solutions to feed the world. AIMS Microbiol..

[13] Pérez-Montaño F., Alías-Villegas C., Bellogín R.A., del Cerro P., Espuny M.R., Jiménez-Guerrero I., López-Baena F.J., Ollero F.J., Cubo T. (2014). Plant growth promotion in cereal and leguminous agricultural important plants: From microorganism capacities to crop production. Microbiol. Res..

[14] Garcia-Fraile P., Menendez E., Rivas R. (2015). Role of bacterial biofertilizers in agriculture and forestry. AIMS Bioeng..

[15] Etesami H., Maheshwari D.K. (2018). Use of plant growth promoting rhizobacteria (PGPRs) with multiple plant growth promoting traits in stress agriculture: Action mechanisms and future prospects. Ecotoxicol. Environ. Saf..

[16] Rahman S.F.S.A., Singh E., Pieterse C.M.J., Schenk P.M. (2018). Emerging microbial biocontrol strategies for plant pathogens. Plant Sci..

[17] Jiménez-Gómez A., Celador-Lera L., Fradejas-Bayón M., Rivas R. (2017). Plant probiotic bacteria enhance the quality of fruit and horticultural crops. AIMS Microbiol..

[18] Stagnari F., Maggio A., Galieni A., Pisante M. (2017). Multiple benefits of legumes for agriculture sustainability: An overview. Chem. Biol. Technol. Agric..

[19] Partida-Martínez L.P., Heil M. (2011). The microbe-free plant: Fact or artifact?. Front. Plant Sci..

[20] Berendsen R.L., Pieterse C.M.J., Bakker P.A.H.M. (2012). The rhizosphere microbiome and plant health. Trends Plant Sci..

[21] Pieterse C.M.J., de Jonge R., Berendsen R.L. (2016). The soil-borne supremacy. Trends Plant Sci..

[22] Zilber-Rosenberg I., Rosenberg E. (2008). Role of microorganisms in the evolution of animals and plants: The hologenome theory of evolution. FEMS Microbiol. Rev..

[23] Bulgarelli D., Schlaeppi K., Spaepen S., Van Themaat E.V.L., Schulze-Lefert P. (2013). Structure and functions of the bacterial microbiota of plants. Annu. Rev. Plant Biol..

[24] Schlaeppi K., Bulgarelli D. (2015). The plant microbiome at work. Mol. Plant Microbe Interact..

[25] Vandenkoornhuyse P., Quaiser A., Duhamel M., Le Van A., Dufresne A. (2015). The importance of the microbiome of the plant holobiont. New Phytol..

[26] Ellis J.G. (2017). Can plant microbiome studies lead to effective biocontrol of plant diseases?. Mol. Plant Microbe Interact..

[27] Busby P.E., Soman C., Wagner M.R., Friesen M.L., Kremer J., Bennett A., Morsy M., Eisen J.A., Leach J.E., Dangl J.L. (2017). Research priorities for harnessing plant microbiomes in sustainable agriculture. PLoS Biol..

[28] Sanchez-Canizares C., Jorrin B., Poole P.S., Tkacz A. (2017). Understanding the holobiont: The interdependence of plants and their microbiome. Curr. Opin. Microbiol..

[29] Hassani M.A., Durán P., Hacquard S. (2018). Microbial interactions within the plant holobiont. Microbiome.

[30] Orozco-Mosqueda M.C., Rocha-Granados M.C., Glick B.R., Santoyo G. (2018). Microbiome engineering to improve biocontrol and plant growth-promoting mechanisms. Microbiol. Res..

[31] Levy A., Conway J.M., Dangl J.L., Woyke T. (2018). Elucidating bacterial gene functions in the plant microbiome. Cell Host Microbe.

[32] Compant S., Samad A., Faist H., Sessitsch A. (2019). A review on the plant microbiome: Ecology, functions and emerging trends in microbial application. J. Adv. Res..

[33] Compant S., Clément C., Sessitsch A. (2010). Plant growth-promoting bacteria in the rhizo-and endosphere of plants: Their role, colonization, mechanisms involved and prospects for utilization. Soil Biol. Biochem..

[34] Martin F.M., Uroz S., Barker D.G. (2017). Ancestral alliances: Plant mutualistic symbioses with fungi and bacteria. Science.

[35] Stone B.W., Weingarten E.A., Jackson C.R. (2018). The role of the phyllosphere microbiome in plant health and function. Annu. Plant Rev..

[36] Hardoim P.R., Van Overbeek L.S., Berg G., Pirttilä A.M., Compant S., Campisano A., Döring M., Sessitsch A. (2015). The hidden world within plants: Ecological and evolutionary considerations for defining functioning of microbial endophytes. Microbiol. Mol. Biol. Rev..

[37] Carvalho T.L.G., Ballesteros H.G.F., Thiebaut F., Ferreira P.C.G., Hemerly A.S. (2016). Nice to meet you: Genetic, epigenetic and metabolic controls of plant perception of beneficial associative and endophytic diazotrophic bacteria in non-leguminous plants. Plant Mol. Biol..

[38] Remans R., Beebe S., Blair M., Manrique G., Tovar E., Rao I., Croonenborghs A., Gutierrez R.T., El-Howeity M., Michiels J. (2008). Physiological and genetic analysis of root responsiveness to auxin-producing plant growth-promoting bacteria in common bean (*Phaseolus vulgaris* L.). Plant Soil.

[39] Ibánẽz F., Angelini J., María T.T., Tonelli L., Fabra A. (2009). Endophytic occupation of peanut root nodules by opportunistic Gammaproteobacteria. Syst. Appl. Microbiol..

[40] Martínez-Hidalgo P., Hirsch A.M. (2017). The nodule microbiome: N2-fixing rhizobia do not live alone. Phytobiomes.

[41] Velázquez E., Carro L., Flores-Félix J.D., Martínez-Hidalgo P., Menéndez E., Ramírez-Bahena M.H., Mulas R., Gonzalez-Andres F., Martinez-Molina E., Peix A., Kumar V., Shivesh M.K., Prasad S. (2017). The legume nodule microbiome: A source of plant growth-promoting bacteria. Probiotics and Plant Health.

[42] Martínez-Hidalgo P., Maymon M., Pule-Meulenberg F., Hirsch A.M. (2019). Engineering root microbiomes for healthier crops and soils using beneficial, environmentally safe bacteria. Can. J. Microbiol..

[43] Downie J.A. (2014). Legume nodulation. Curr. Biol..

[44] Ferguson B.J., Mens C., Hastwell A.H., Zhang M., Su H., Jones C.H., Chu X., Gresshoff P.M. (2019). Legume nodulation: The host controls the party. Plant Cell Environ..

[45] Vargas L.K., Volpiano C.G., Lisboa B.B., Giongo A., Beneduzi A., Passaglia L.M.P., Zaidi A., Khan M., Musarrat J. (2017). Potential of rhizobia as plant growth-promoting rhizobacteria. Microbes for Legume Improvement.

[46] Garrido-Oter R., Nakano R.T., Dombrowski N., Ma K.W., McHardy A.C., Schulze-Lefert P., The AgBiome Team (2018). Modular traits of the rhizobiales root microbiota and their evolutionary relationship with symbiotic Rhizobia. Cell Host Microbe.

[47] Das K., Prasanna R., Saxena A.K. (2017). Rhizobia: A potential biocontrol agent for soilborne fungal pathogens. Folia Microbiol..

[48] Aeron A., Maheshwari D.K., Dheeman S., Agarwal M., Dubey R.C., Bajpai V.K. (2017). Plant growth promotion and suppression of charcoal-rot fungus (*Macrophomina phaseolina*) in velvet bean (*Mucuna pruriens* L.) by root nodule bacteria. J. Phytopathol..

[49] Jack C.N., Wozniak K.J., Porter S.S., Friesen M.L. (2019). Rhizobia protect their legume hosts against soil-borne microbial antagonists in a host-genotype-dependent manner. Rhizosphere.

[50] Yeoh Y.K., Dennis P.G., Paungfoo-Lonhienne C., Weber L., Brackin R., Ragan M.A., Schmidt S., Hugenholtz P. (2017). Evolutionary conservation of a core root microbiome across plant phyla along a tropical soil chronosequence. Nat. Commun..

[51] Banerjee S., Schlaeppi K., van der Heijden M.G.A. (2018). Keystone taxa as drivers of microbiome structure and functioning. Nat. Rev. Microbiol..

[52] Yanni Y.G., Rizk R.Y., Corich V., Squartini A., Ninke K., Philip-Hollingsworth S., Orgambide G., de Bruijn F.J., Stoltzfus J., Buckley D. (1997). Natural endophytic association between *R. legumionosarum* bv. trifolli and rice root and assessment of its potential to promote rice growth. Plant Soil.

[53] Peix A., Rivas-Boyero A.A., Mateos P.F., Rodriguez-Barrueco C., Martınez-Molina E., Velazquez E. (2001). Growth promotion of chickpea and barley by a phosphate solubilizing strain of *Mesorhizobium mediterraneum* under growth chamber conditions. Soil Biol. Biochem..

[54] Matiru V.N., Jaffer M.A., Dakora F.D. (2005). Rhizobial infection of African landraces of sorghum (*Sorghum bicolor* L.) and finger millet (*Eleucine coracana* L.) promotes plant growth and alters tissue nutrient concentration under axenic conditions. Symbiosis.

[55] García-Fraile P., Carro L., Robledo M., Ramírez-Bahena M.H., Flores-Félix J.D., Fernández M.T., Mateos P.F., Rivas R., Igual J.M., Martínez-Molina E. (2013). *Rhizobium* promotes non-legumes growth and quality in several production steps: Towards a biofertilization of edible raw vegetables healthy for humans. PLoS ONE.

[56] Flores-Félix J.D., Menéndez E., Rivera L.P., Marcos-García M., Martínez-Hidalgo P., Mateos P.F., Martínez-Molina E., Velázquez E., García-Fraile P., Rivas R. (2013). Use of *Rhizobium leguminosarum* as a potential biofertilizer for *Lactuca sativa* and *Daucus carota* crops. J. Plant Nutr. Soil Sci..

[57] Flores-Félix J.D., Marcos-García M., Silva L.R., Menéndez E., Martínez-Molina E., Mateos P.F., Velázquez E., García-Fraile P., Andrade P., Rivas R. (2015). *Rhizobium* as plant probiotic for strawberry production under microcosm conditions. Symbiosis.

[58] Yanni Y.G., Dazzo F.B., Squartini A., Zanardo M., Zidan M.I., Elsadany A.E.Y. (2016). Assessment of the natural endophytic association between Rhizobium and wheat and its ability to increase wheat production in the Nile delta. Plant Soil.

[59] Jiménez-Gómez A., Flores-Félix J.D., García-Fraile P., Mateos P.F., Menéndez E., Velázquez E., Rivas R. (2018). Probiotic activities of *Rhizobium laguerreae* on growth and quality of spinach. Sci. Rep..

[60] Qureshi M.A., Shahzad H., Saeed M.S., Ullah S., Ali M.A., Mujeeb F., Anjum M.A. (2019). Relative potential of rhizobium species to enhance the growth and yield attributes of cotton (*Gossypium hirsutum* L.). Eurasian J. Soil Sci..

[61] Cassán F., Diaz-Zorita M. (2016). *Azospirillum* sp. in current agriculture: From the laboratory to the field. Soil Biol. Biochem..

[62] Korir H., Mungai N.W., Thuita M., Hamba Y., Masso C. (2017). Co-inoculation effect of rhizobia and plant growth promoting rhizobacteria on common bean growth in a low phosphorus soil. Front. Plant Sci..

[63] Brígido C., Singh S., Menéndez E., Tavares M.J., Glick B.R., Félix M.D.R., Oliveira S., Carvalho M. (2019). Diversity and functionality of culturable endophytic bacterial communities in chickpea plants. Plants.

[64] Trujillo M.E., Alonso-Vega P., Rodríguez R., Carro L., Cerda E., Alonso P., Martínez-Molina E. (2010). The genus *Micromonospora* is widespread in legume root nodules: The example of *Lupinus angustifolius*. ISME J..

[65] Muresu R., Polone E., Sulas L., Baldan B., Tondello A., Delogu G., Cappuccinelli P., Alberghini S., Benhizia Y., Benhizia H. (2008). Coexistence of predominantly nonculturable rhizobia with diverse, endophytic bacterial taxa within nodules of wild legumes. FEMS Microbiol. Ecol..

[66] De Meyer S.E., De Beuf K., Vekeman B., Willems A. (2015). A large diversity of non-rhizobial endophytes found in legume root nodules in Flanders (Belgium). Soil Biol. Biochem..

[67] Hartman K., van der Heijden M.G., Roussely-Provent V., Walser J.C., Schlaeppi K. (2017). Deciphering composition and function of the root microbiome of a legume plant. Microbiome.

[68] Edwards J., Johnson C., Santos-Medellín C., Lurie E., Podishetty N.K., Bhatnagar S., Eisen J.A., Sundaresan V. (2015). Structure, variation, and assembly of the root-associated microbiomes of rice. Proc. Natl. Acad. Sci. USA.

[69] Niu B., Paulson J.N., Zheng X., Kolter R. (2017). Simplified and representative bacterial community of maize roots. Proc. Natl. Acad. Sci. USA.

[70] Gyaneshwar P., Hirsch A.M., Moulin L., Chen W.M., Elliott G.N., Bontemps C., Estrada-de los Santos P., Gross E., Bueno dos Reis F., Sprent J. (2011). Legume-nodulating betaproteobacteria: Diversity, host range, and future prospects. Mol. Plant Microbe Interact..

[71] Hu J., Wei Z., Weidner S., Friman V.P., Xu Y.C., Shen Q.R., Jousset A. (2017). Probiotic *Pseudomonas* communities enhance plant growth and nutrient assimilation via diversity-mediated ecosystem functioning. Soil Biol. Biochem..

[72] Shiraishi A., Matsushita N., Hougetsu T. (2010). Nodulation in black locust by the Gammaproteobacteria *Pseudomonas* sp. and the Betaproteobacteria *Burkholderia* sp. Syst. Appl. Microbiol..

[73] Latif S., Khan S., Naveed M., Mustafa G., Bashir T., Mumtaz A.S. (2013). The diversity of Rhizobia, Sinorhizobia and novel non-Rhizobial *Paenibacillus* nodulating wild herbaceous legumes. Arch. Microbiol..

[74] González A.H., Morales Londoño D., Pille da Silva E., Nascimento F.X.I., de Souza L.F., da Silva B.G., Canei A.D., de Armas R.D., Giachini A.J., Soares C.R.F.S. (2019). *Bradyrhizobium* and *Pseudomonas* strains obtained from coal-mining areas nodulate and promote the growth of *Calopogonium muconoides* plants used in the reclamation of degraded areas. J. Appl. Microbiol..

[75] Peix A., Ramírez-Bahena M.H., Velázquez E., Bedmar E.J. (2015). Bacterial associations with legumes. Crit. Rev. Plant Sci..

[76] Santoyo G., Moreno-Hagelsieb G., del Carmen Orozco-Mosqueda M., Glick B.R. (2016). Plant growth-promoting bacterial endophytes. Microbiol. Res..

[77] Kandel S., Joubert P., Doty S. (2017). Bacterial endophyte colonization and distribution within plants. Microorganisms.

[78] Rho H., Hsieh M., Kandel S.L., Cantillo J., Doty S.L., Kim S.H. (2018). Do endophytes promote growth of host plants under stress? A meta-analysis on plant stress mitigation by endophytes. Microb. Ecol..

[79] Ku Y.S., Rehman H.M., Lam H.M. (2019). Possible roles of rhizospheric and endophytic microbes to provide a safe and affordable means of crop biofortification. Agronomy.

[80] Jiménez-Gómez A., García-Estévez I., García-Fraile P., Escribano-Bailón M.T., Rivas R. (2020). Increase in phenolic compounds of *Coriandrum sativum* L. after the application of a *Bacillus halotolerans* biofertilizer. J. Sci. Food Agric..

[81] Pastor-Bueis R., Sánchez-Cañizares C., James E.K., González-Andrés F. (2019). Formulation of a highly effective inoculant for common bean based on an autochthonous elite strain of *Rhizobium leguminosarum* bv. phaseoli, and genomic-based insights into its agronomic performance. Front. Microbiol..

[82] Flores-Félix J.D., Velázquez E., García-Fraile P., González-Andrés F., Silva L.R., Rivas R. (2018). *Rhizobium* and *Phyllobacterium* bacterial inoculants increase bioactive compounds and quality of strawberries cultivated in field conditions. Food Res. Int..

[83] Chiboub M., Jebara S.H., Abid G., Jebara M. (2019). Co-inoculation effects of Rhizobium sullae and Pseudomonas sp. on growth, antioxidant status, and expression pattern of genes associated with heavy metal tolerance and accumulation of cadmium in *Sulla coronaria*. J. Plant Growth Regul..

[84] Kaul S., Sharma T., Dhar M.K. (2016). “Omics” tools for better understanding the plant-endophyte interactions. Front. Plant Sci..

[85] Plett J.M., Martin F.M. (2018). Know your enemy, embrace your friend: Using omics to understand how plants respond differently to pathogenic and mutualistic microorganisms. Plant J..

[86] Finkel O.M., Castrillo G., Paredes S.H., González I.S., Dangl J.L. (2017). Understanding and exploiting plant beneficial microbes. Curr. Opin. Plant Biol..

[87] Samavat S., Samavat S., Mafakheri S., Shakouri M.J. (2012). Promoting common bean growth and nitrogen fixation by the co-inoculation of *Rhizobium* and *Pseudomonas fluorescens* isolates. Bulg. J. Agric. Sci..

[88] Verma J.P., Yadav J., Tiwari K.N. (2010). Application of *Rhizobium* sp. BHURC01 and plant growth promoting rhizobacteria on nodulation, plant biomass and yields of chickpea (*Cicer arietinum* L.). Int. J. Agric. Res..

[89] Verma J.P., Yadav J., Tiwari K.N. (2012). Enhancement of nodulation and yield of chickpea by co-inoculation of indigenous *Mesorhizobium* spp. and plant growth–promoting rhizobacteria in Eastern Uttar Pradesh. Commun. Soil Sci. Plant Anal..

[90] Egamberdieva D., Wirth S.J., Shurigin V.V., Hashem A., Abd_Allah E.F. (2017). Endophytic bacteria improve plant growth, symbiotic performance of chickpea (*Cicer arietinum* L.) and induce suppression of root rot caused by *Fusarium solani* under salt stress. Front. Microbiol..

[91] Fukami J., de la Osa C., Ollero F.J., Megías M., Hungria M. (2018). Co-inoculation of maize with *Azospirillum brasilense* and *Rhizobium tropici* as a strategy to mitigate salinity stress. Funct. Plant Biol..

[92] Halverson L.J., Handelsman J. (1991). Enhancement of soybean nodulation by *Bacillus cereus* UW85 in the field and in a growth chamber. Appl. Environ. Microbiol..

[93] Pandey P., Maheshwari D.K. (2007). Bioformulation of *Burkholderia* sp. MSSP with a multispecies consortium for growth promotion of *Cajanus cajan*. Can. J. Microbiol..

[94] Mishra P.K., Mishra S., Selvakumar G., Bisht J.K., Kundu S., Gupta H.S. (2009). Co-inoculation of *Bacillus thuringeinsis* -KR1 with *Rhizobium leguminosarum* enhances plant growth and nodulation of pea (*Pisum sativum* L.) and lentil (*Lens culinaris* L.). World J. Microbiol. Biotechnol..

[95] Tariq M., Hameed S., Yasmeen T., Ali A. (2012). Non-rhizobial bacteria for improved nodulation and grain yield of mung bean [*Vigna radiata* (L.) Wilczek]. Afr. J. Biotechnol..

[96] Masciarelli O., Llanes A., Luna V. (2014). A new PGPR co-inoculated with *Bradyrhizobium japonicum* enhances soybean nodulation. Microbiol. Res..

[97] Diez-Mendez A., Menéndez E., García-Fraile P., Celador-Lera L., Rivas R., Mateos P.F. (2015). *Rhizobium cellulosilyticum* as a co-inoculant enhances *Phaseolus vulgaris* grain yield under greenhouse conditions. Symbiosis.

[98] Prakamhang J., Tittabutr P., Boonkerd N., Teamtisong K., Uchiumi T., Abe M., Teaumroong N. (2015). Proposed some interactions at molecular level of PGPR coinoculated with *Bradyrhizobium diazoefficiens* USDA110 and *B. japonicum* THA6 on soybean symbiosis and its potential of field application. Appl. Soil Ecol..

[99] Lu J., Yang F., Wang S., Ma H., Liang J., Chen Y. (2017). Co-existence of rhizobia and diverse non-rhizobial bacteria in the rhizosphere and nodules of *Dalbergia odorifera* seedlings inoculated with *Bradyrhizobium elkanii*, *Rhizobium multihospitium*—Like and *Burkholderia pyrrocinia*—Like Strains. Front. Microbiol..

[100] Bona E., Cantamessa S., Massa N., Manassero P., Marsano F., Copetta A., Lingua G., D’Agostino G., Gamalero E., Berta G. (2017). Arbuscular mycorrhizal fungi and plant growth-promoting pseudomonads improve yield, quality and nutritional value of tomato: A field study. Mycorrhiza.

[101] Raklami A., Bechtaoui N., Tahiri A., Anli M., Meddich A., Oufdou K. (2019). Use of rhizobacteria and mycorrhizae consortium in the open field as a strategy for improving crop nutrition, productivity and soil fertility. Front. Microbiol..

[102] Dal Cortivo C., Barion G., Ferrari M., Visioli G., Dramis L., Panozzo A., Vamerali T. (2018). Effects of field inoculation with VAM and bacteria consortia on root growth and nutrients uptake in common wheat. Sustainability.

[103] Walker V., Couillerot O., Felten A.V., Bellvert F., Jansa J., Maurhofer M., Bally R., Moënne-Loccoz Y., Comte G. (2012). Variation of secondary metabolite levels in maize seedling roots induced by inoculation with *Azospirillum, Pseudomonas* and *Glomus* consortium under field conditions. Plant Soil.

[104] Hungria M., Nogueira M.A., Silva Araujo R.S. (2013). Co-inoculation of soybeans and common beans with rhizobia and azospirilla: Strategies to improve sustainability. Biol. Fertil. Soils.

[105] Elkoca E., Turan M., Donmez M.F. (2010). Effects of single, dual and triple inoculations with *Bacillus subtilis*, *Bacillus megaterium* and *Rhizobium leguminosarum* bv. phaseoli on nodulation, nutrient uptake, yield and yield parameters of common bean (*Phaseolus vulgaris* L. cv. ‘Elkoca-05’). J. Plant Nutr..

[106] Kumar P., Pandey P., Dubey R.C., Maheshwari D.K. (2010). Bacteria consortium optimization improves nutrient uptake, nodulation, disease suppression and growth of the common bean (*Phaseolus vulgaris*) in both pot and field studies. Rhizosphere.

[107] Singh N., Singh G., Aggarwal N., Khanna V. (2018). Yield enhancement and phosphorus economy in lentil (*Lens culinaris* Medikus) with integrated use of phosphorus, *Rhizobium* and plant growth promoting rhizobacteria. J. Plant Nutr..

[108] Kumawat K.C., Sharma P., Sirari A., Singh I., Gill B.S., Singh U., Saharan K. (2019). Synergism of *Pseudomonas aeruginosa* (LSE-2) nodule endophyte with *Bradyrhizobium* sp. (LSBR-3) for improving plant growth, nutrient acquisition and soil health in soybean. World J. Microbiol. Biotechnol..

[109] Verma J.P., Yadav J., Tiwari K.N., Kumar A. (2013). Effect of indigenous *Mesorhizobium* spp. and plant growth promoting rhizobacteria on yields and nutrients uptake of chickpea (*Cicer arietinum* L.) under sustainable agriculture. Ecol. Eng..

[110] Lally R.D., Galbally P., Moreira A.S., Spink J., Ryan D., Germaine K.J., Dowling D.N. (2017). Application of endophytic *Pseudomonas fluorescens* and a bacterial consortium to *Brassica napus* can increase plant height and biomass under greenhouse and field conditions. Front. Plant Sci..

[111] Lucas J.A., Ramos-Solano B., Montes F., Ojeda J., Megias M., Gutierrez Mañero F.J. (2009). Use of two PGPR strains in the integrated management of blast disease in rice (*Oryza sativa*) in Southern Spain. Field Crops Res..

[112] Barra P.J., Inostroza N.G., Mora M.L., Crowley D.E., Jorquera M.A. (2017). Bacterial consortia inoculation mitigates the water shortage and salt stress in an avocado (*Persea americana* Mill.) nursery. Appl. Soil Ecol..

[113] Khan Z., Kandel S., Ramos D., Ettl G., Kim S.H., Doty S. (2015). Increased biomass of nursery-grown Douglas-fir seedlings upon inoculation with diazotrophic endophytic consortia. Forest.

[114] Kumar H., Dubey R.C., Maheshwari D.K. (2017). Seed-coating fenugreek with *Burkholderia* rhizobacteria enhances yield in field trials and can combat *Fusarium* wilt. Rhizosphere.

[115] Imran A., Mirza M.S., Shah T.M., Malik K.A., Hafeez F.Y. (2015). Differential response of kabuli and desi chickpea genotypes toward inoculation with PGPR in different soils. Front. Microbiol..

[116] Yanni Y.G., Dazzo F.B. (2010). Enhancement of rice production using endophytic strains of *Rhizobium leguminosarum* bv. trifolii in extensive field inoculation trials within the Egypt Nile delta. Plant Soil.

[117] He Y., Pantigoso H.A., Wu Z., Vivanco J.M. (2019). Co-inoculation of *Bacillus* sp. and *Pseudomonas* putida at different development stages acts as a biostimulant to promote growth, yield and nutrient uptake of tomato. J. Appl. Microbiol..

[118] Qiao J., Yu X., Liang X., Liu Y., Borriss R., Liu Y. (2017). Addition of plant-growth-promoting *Bacillus subtilis* PTS-394 on tomato rhizosphere has no durable impact on composition of root microbiome. BMC Microbiol..

[119] Camacho M., Santamaría C., Temprano F., Rodriguez-Navarro D.N., Daza A. (2001). Co-inoculation with *Bacillus* sp. CECT 450 improves nodulation in *Phaseolus vulgaris* L. Can. J. Microbiol..

[120] López-Mondéjar R., Kostovčík M., Lladó S., Carro L., García-Fraile P. (2017). Exploring the plant microbiome through multi-omics approaches. Probiotics in Agroecosystem.

[121] Martiny A.C. (2019). High proportions of bacteria are culturable across major biomes. ISME J..

[122] Steen A.D., Crits-Christoph A., Carini P., DeAngelis K.M., Fierer N., Lloyd K.G., Thrash J.C. (2019). High proportions of bacteria and archaea across most biomes remain uncultured. ISME J..

[123] Kong Z., Hart M., Liu H. (2018). Paving the way from the lab to the field: Using synthetic microbial consortia to produce high-quality crops. Front. Plant Sci..

[124] Sessitsch A., Pfaffenbichler N., Mitter B. (2019). Microbiome applications from lab to field: Facing complexity. Trends Plant Sci..

[125] Bashan Y., Kloepper J.W., de-Bashan L.E., Nannipieri P. (2016). A need for disclosure of the identity of microorganisms, constituents, and application methods when reporting tests with microbe-based or pesticide-based products. Biol. Fertil. Soils.

[126] Malusá E., Vassilev N. (2014). A contribution to set a legal framework for biofertilisers. Appl. Microbiol. Biotechnol..

[127] Barquero M., Pastor-Buies R., Urbano B., González-Andrés F., Zúñiga-Dávila D., González-Andrés F., Ormeño-Orrillo E. (2019). Challenges, regulations and future actions in biofertilizers in the european agriculture: From the lab to the field. Microbial Probiotics for Agricultural Systems. Sustainability in Plant and Crop Protection.

[128] Kaminsky L.M., Trexler R.V., Malik R.J., Hockett K.L., Bell T.H. (2019). The inherent conflicts in developing soil microbial inoculants. Trends Biotechnol..

[129] Gadhave K.R., Devlin P.F., Ebertz A., Ross A., Gange A.C. (2018). Soil inoculation with *Bacillus* spp. modifies root endophytic bacterial diversity, evenness, and community composition in a context-specific manner. Microb. Ecol..

[130] Ambrosini A., de Souza R., Passaglia L.M. (2016). Ecological role of bacterial inoculants and their potential impact on soil microbial diversity. Plant Soil.

[131] Jha P.N., Gomaa A.B., Yanni Y.G., El-Saadany A.E.Y., Stedtfeld T.M., Stedtfeld R.D., Gantner S., Cole B.C.J., Hashsham S.A., Dazzo F.B. (2019). Alterations in the endophyte-enriched root-associated microbiome of rice receiving growth-promoting treatments of urea fertilizer and *Rhizobium* biofertilizer. Microb. Ecol..

